# Transcription Factors Sox2 and Sox3 Directly Regulate the Expression of Genes Involved in the Onset of Oligodendrocyte Differentiation

**DOI:** 10.3390/cells13110935

**Published:** 2024-05-29

**Authors:** Jesse Rupprecht, Simone Reiprich, Tina Baroti, Carmen Christoph, Elisabeth Sock, Franziska Fröb, Michael Wegner

**Affiliations:** Institut für Biochemie, Friedrich-Alexander-Universität Erlangen-Nürnberg, 91054 Erlangen, Germany

**Keywords:** transcription factor, SoxB1 proteins, myelin, glia, Zfp488, Nkx2.2

## Abstract

Rapid information processing in the central nervous system requires the myelination of axons by oligodendrocytes. The transcription factor Sox2 and its close relative Sox3 redundantly regulate the development of myelin-forming oligodendrocytes, but little is known about the underlying molecular mechanisms. Here, we characterized the expression profile of cultured oligodendroglial cells during early differentiation and identified Bcas1, Enpp6, Zfp488 and Nkx2.2 as major downregulated genes upon Sox2 and Sox3 deletion. An analysis of mice with oligodendrocyte-specific deletion of Sox2 and Sox3 validated all four genes as downstream targets in vivo. Additional functional assays identified regulatory regions in the vicinity of each gene that are responsive to and bind both Sox proteins. Bcas1, Enpp6, Zfp488 and Nkx2.2 therefore likely represent direct target genes and major effectors of Sox2 and Sox3. Considering the preferential expression and role of these genes in premyelinating oligodendrocytes, our findings suggest that Sox2 and Sox3 impact oligodendroglial development at the premyelinating stage with Bcas1, Enpp6, Zfp488 and Nkx2.2 as their major effectors.

## 1. Introduction

Oligodendrocytes are myelin-forming cells of the vertebrate nervous system and are essential for rapid saltatory conduction in the central nervous system (CNS). They develop from neuroepithelial precursor cells of the ventricular zone that also give rise to neurons and astrocytes. After specification to oligodendroglial progenitor cells (OPCs) and further expansion of the OPC pool, cells migrate to their destination site where they develop into premyelinating oligodendrocytes and initiate the terminal differentiation process that eventually converts them into mature oligodendrocytes with myelin sheaths.

This multistage process of developmental myelination is regulated by external signals in combination with a cell-intrinsic gene regulatory network that consists of many interacting regulators, including transcription factors, chromatin remodeling and modifying proteins, as well as regulatory RNAs [1,2,3,4]. Remyelination in the adult CNS follows similar principles.

Over the years, several members of the Sox protein family have been identified as transcription factors with prominent roles in oligodendroglial cells. This includes Sox10 as an important determinant of oligodendroglial identity that is active at all stages of oligodendrocyte development and is particularly relevant for differentiation and myelination [5,6]. Sox10 is aided in its task by the closely related, paralogous Sox8 and Sox9, and modulated in its function by the more distantly related Sox5 and Sox6 proteins [7,8]

Another Sox protein with importance in oligodendroglial cells is Sox2. Despite early in vitro-based reports on the absence of Sox2 in oligodendroglial cells [9], it is clear now that Sox2 is expressed in cells of the oligodendrocyte lineage in vivo [10,11]. This includes both OPCs and premyelinating oligodendrocytes. At later stages of terminal differentiation, Sox2 expression is extinguished. Depending on the Cre transgene used for the deletion of Sox2 in oligodendroglial cells, the exact CNS region studied, the time point and the physiological context (e.g., developmental myelination vs. remyelination), slightly varying results have been obtained regarding its function [11,12,13,14]. However, the synopsis of all studies suggests a role of Sox2 in OPCs where it sustains progenitor cell characteristics and permits efficient proliferation and proper expansion of the cell pool, as well as a second function in the recruitment of OPCs for terminal differentiation and in priming them as premyelinating oligodendrocytes for the myelination process. Intriguingly, there is also evidence that Sox2 is co-expressed at all times during oligodendrocyte development with another member of the SoxB1 subgroup of Sox proteins, the closely related Sox3 [15]. Both proteins function redundantly, with Sox2 being the bigger contributor to the shared function [11].

Here, we analyze the consequences of a joint loss of Sox2 and Sox3 on the oligodendroglial expression profile and detect a decreased expression of genes that are typical for the premyelinating stage. By focusing on the role of Sox2 and Sox3 in the early phases of differentiation, we furthermore show that Bcas1, Enpp6, Zfp488 and Nkx2.2 are direct targets of Sox2 and Sox3 in vitro as well as in vivo. We therefore conclude that Sox2 and Sox3 exert their effects on oligodendroglial differentiation in premyelinating oligodendrocytes at least in part through these molecules. From the fact that the exact regulatory regions used by Sox2 and Sox3 to activate the expression of these genes overlap only partially with regulatory regions employed by Sox10 and/or Olig2, we furthermore infer that the molecular mode of action differs at least partially between SoxB1 proteins and other transcription factors with relevance for oligodendrocyte differentiation.

## 2. Materials and Methods

### 2.1. Plasmids

Expression plasmids were based on pCMV5 for Sox10, Sox2, Nkx2.2 and Myrf, as described [11,16,17]. Additionally, the Sox3 coding sequence (BC052024.1) was inserted into pCMV5 using EcoRI and XbaI restriction enzymes. Reporter plasmids were based on pTATA-luc [16,17] and contained potential regulatory regions in front of β-globin minimal promoters and luciferase reporters. For *Nkx2.2* regulatory regions, the plasmids have been described before [17]. For *Bcas1*, *Enpp6* and *Zfp488* genes, potential regulatory regions were identified from published ChiP-seq data for Sox10, Sox2 and Olig2 (GSE64703, GSE35496, GSE42447), amplified from mouse genomic DNA by PCR and inserted between KpnI and XhoI sites of pTATA-luc. Coordinates for the analyzed regions are as follows: for the *Bcas1* locus −40RR (mm10, chr2:170,469,729–170,469,922), −3.2RR (mm10, chr2:170,431,095–170,431,255), +20RR (mm10, chr2:170,406,423–170,407,031) and +23RR (mm10, chr2:170,403,672–170,404,285); for the *Enpp6* locus −87RR (mm10, chr8:46,896,077–46,897,551), −40RR (mm10, chr8:46,945,487–46,946,631), −18RR (mm10, chr8:46,968,408–46,968,577), +1.5RR (mm10, chr8:46,988,412–46,989,034), +18RR (mm10, chr8:47,005,420–47,006,129), +43RR (mm10, chr8:47,030,633–47,031,002), +69RR (mm10, chr8:47,056,707–47,057,098) and +151RR (mm10, chr8:47,138,031–47,139,054), and for the *Zfp488* locus −1.6RR (mm10, chr14:33,976,737–33,977,235) and the promoter (mm10, chr14:33,974,752–33,975,391).

### 2.2. Cell Lines, Culture Conditions, and Luciferase Assays

Neuro2a cells were obtained from ATCC (CCl-131) and maintained in high-glucose Gibco DMEM (Thermo Fisher Scientific, Dreieich, Germany, 11965092) with 10% Gibco FCS (Thermo Fisher Scientific, 10500064) and Pen/Strep (Thermo Fisher Scientific, 15140122). For luciferase reporter assays, Neuro2a cells were seeded in 24-well plates and transfected with 1.5 µg of luciferase reporter plasmid and 750 ng of pCMV5-based expression plasmids for *Nkx2.2* regulatory regions, and with 300 ng luciferase reporter plasmid and 300 ng pCMV5-based expression plasmids for *Bcas1*, *Enpp6* and *Zfp488* regulatory regions using Xfect according to the manufacturer’s instructions (Takara Biotech, Saint-Germain-en-Laye, France, 631318). Overall, the DNA amounts per well were kept constant. Luciferase assays and their analysis were reported elsewhere [17].

### 2.3. Animals, Immunohistochemistry, In Situ Hybridization and Primary Oligodendroglial Cultures

Rats and mice were housed under standard conditions with 12 h dark–light cycles and continuous access to food and water according to animal welfare laws. Both male and female pups were used for analysis. Transgenic and control mice were of a mixed C3H x C57Bl/6J background. Transgenic mice carried floxed *Sox2* [18] and *Sox3* [19] alleles for some experiments in combination with a Sox10-Cre BAC transgene [20].

For immunochemistry, spinal cord tissue from newborn pups of Sox2^flox/flox^ Sox3^flox/flox^ Sox10-Cre mice and controls underwent fixation in 4% paraformaldehyde, dehydration in 30% sucrose, freezing and tissue sectioning on a cryotome. Transverse spinal cord sections (10 µm thickness) at forelimb level were stained with primary antibodies against Sox10 (goat antiserum; 1:3000) [21], Bcas1 (rabbit antiserum; 1:500; Synaptic System, Göttingen, Germany; Cat. No. 445003) and Nkx2.2 (mouse antiserum; 1:50; DS Hybridoma Bank, University of Iowa, Iowa City, IA, USA; 74.5A5). Secondary antibodies conjugated to Alexa Fluor 488 (Molecular Probes, Eugene, OR, USA) or Cy3 immunofluorescent dyes (Dianova, Hamburg, Germany) were used for detection. Adjacent cryotome sections were also used for in situ hybridizations using DIG-labeled antisense riboprobes specific for *Enpp6* (nucleotides 591-1199 of NM_177304) and *Zfp488* (complete open reading frame of NM_001013777.2) following standard procedures [6]. Staining was documented on a Leica DMI6000B inverse microscope (Leica, Wetzlar, Germany) or Leica MZFLIII stereomicroscope equipped with camera.

To obtain rodent primary oligodendroglial cells, mixed glial cultures were prepared from the brains of newborn Wistar rats or the brains of Sox2^flox/flox^ Sox3^flox/flox^ mouse pups two days after birth [11,22,23]. Following shake-off of the loosely attached oligodendroglial cells from the astrocytic cell layer and microglial depletion, primary rat oligodendroglial cells were grown on poly-ornithine (Sigma-Aldrich, Hamburg, Germany, P3655)-coated cell culture dishes in N2-supplemented medium containing 10 ng/mL PDGF-A and 10 ng/mL FGF2 to allow for proliferation. For differentiation, growth factor-containing medium was replaced by SATO medium containing T3 and T4 hormones. Mouse primary oligodendroglial cells were seeded onto dishes coated with poly-D-lysine (Sigma-Aldrich, P6407) and laminin (Sigma-Aldrich, L2020). Proliferation was in basal medium supplemented with B27, N2, ITS, 10 ng/mL PDFG-AA and 10 ng/mL FGF2. To induce recombination of the floxed alleles, 0.4 pmol/mL Tat-Cre protein was added 24 h and 48 h after seeding. Differentiation was induced after 3 days in culture by exposure to 1% FCS for an additional 4 days before harvest.

### 2.4. RNA Preparation, and Quantitative RT-PCR

Total RNA was isolated from primary mouse oligodendroglial cells after 4 days of differentiation using the miRVana miRNA isolation kit (Ambion, Thermo Fisher Scientific) and DNAse-treated. To determine the efficiency of Cre-dependent gene deletion, RNA samples were reverse transcribed and subjected to quantitative PCR (CFX96 Real-Time PCR System, Bio Rad, Feldkirchen, Germany) using primers 5′-AGACTCCGGGCGATGAAAA-3′ and 5′-TGCAGAATCAAAACCCAGCAA-3′ for *Sox2*. Transcript levels were normalized to *Rpl8*.

### 2.5. RNA-Sequencing (RNA-Seq) and Bioinformatical Analysis

RNA samples from primary mouse oligodendroglial cells were quantified on a Qubit 4.0 Fluorometer (Life Technologies, Thermo Fisher Scientific) and RNA integrity confirmed on an Agilent 5300 Fragment Analyzer (Agilent Technologies, Santa Clara, CA, USA). Sequencing libraries were prepared using the TruSeq Stranded mRNA Kit (Illumina, San Diego, CA, USA), multiplexed and sequenced on an Illumina HiSeq2500 platform with a 2× 100 bp single-end configuration. On average, 59 million reads were generated per sample library and aligned to the Mus musculus GRCm38 genome. To reduce batch effects, RuvSeq (Galaxy Version 1.26.0) was applied before DESeq2 (Galaxy Version 2.11.40.8) was used to generate PCA plots and identify genes in samples from Sox2/Sox3 double-deficient oligodendroglial cells that are differentially expressed relative to controls. Differentially expressed genes (DEG) were defined by a log2-fold change ≥ ±0.7 and a *p*-value ≤ 0.05. Gene expression datasets are deposited in GEO under GSE263925. Gene ontology (GO) analysis (DAVID) [24] and gene set enrichment analysis (GSEA, Broad Institute, http://software.broadinstitute.org/gsea/index.jsp (accessed on 10 February 2024)) were used to identify changes in the expression of select gene sets.

### 2.6. Chromatin Immunoprecipitation (ChIP)

Chromatin immunoprecipitations were carried out as published [17]. The chromatin of primary rat oligodendroglia, which was cross-linked, purified and sheared directly after shake-off (OPC) or after 4 days of differentiation (OL), was precipitated with self-made rabbit anti-Sox2 antiserum [11] and the corresponding rabbit pre-immune serum coupled to Protein A-Sepharose beads (GE Healthcare, Düsseldorf, Germany, 17-0780-01) pre-blocked with bovine serum albumin. After cross-link reversal and purification from input and precipitated chromatin fragments, DNA was used for quantitative PCR to calculate the fold enrichment of specific DNA regions with anti-Sox2 antibodies over the enrichment with pre-immune serum using the ΔΔCt method. The following primers were used: 5′-GAATGACTGAAATCCGGCGC-3′ and 5′-GGAGAAAGGGAACGGCTTGA-3′ for *Bcas1* −40RR, 5′-GTGTGAAAGGGTGGTGAGCA-3′ and 5′-GCCATTTCTCTGCCCCATTACT-3′ for *Bcas1* −3.2RR, 5′- TGGGGTTTTATCTGGAGCCG-3′ and 5′-TCTTGACACTCTCTCGGCCT-3′ for *Bcas1* +20RR, 5′-GGAAGGACCCTGAGAGAAACA-3′ and 5′-ATGGACAAGCGGGGCATTTA-3′ for *Bcas1* +23RR, 5′-GGGTCTGTATTAAAGGTGCTGC-3′ and 5′-CTCAGAGATTTCCCCCTGCC-3′ for *Enpp6* −87RR, 5′-GAGGGACAAAAGGAAGGGGG-3′ and 5′-GTCGGCCAAAACAGAGAGGA-3′ for *Enpp6* −40RR, 5′-TCCAAAGTCAAGACCATAACTCTGA-3′ and 5′-TGGTCTGCTTGGAACCGAAG-3′ for *Enpp6* −18RR, 5′-TGACAGAGCCTTTGTGCCAA-3′ and 5′-GGAGACACAATTCATAAGCTGCC-3′ for *Enpp6* +1.5RR, 5′-GGGGTTGGAGTTGGGGAG-3′ and 5′-TCAAATGCAATCACAGGAACCA-3′ for *Enpp6* +18RR, 5′-AGTATGGTGGCTTCTCAAGGA-3′ and 5′-CTGCTTCTTGTTTGTCCTTCTCTG-3′ for *Enpp6* +43RR, 5′-GTCAAGGAAGGGCAAGGACA-3′ and 5′-GGCAATCCTCGTCCTTCCTT-3′ for *Enpp6* +151RR, 5′-GCTGCTCAACAAGGCCCTT-3′ and 5′-AATGTCGATGGCAAAGGGGG-3′ for *Zfp488* −1.6RR, and 5′-TCGCACAAACATGGCTCAGA-3′ and 5′-CCAATGGCAGAAAAGATGGGC-3′ for *Zfp488* promotor. Primers for the detection of *Nkx2.2* regulatory regions and negative controls (neg1, neg2) were described before [17].

### 2.7. Statistical Analysis

Biological replicates were defined as the results from independent animals, experiments or independently generated, separate samples. Sample size was *n* = 2 for the RNA-seq and *n* ≥ 3 for all other mice and molecular biology experiments. Investigators were not blinded in animal experiments. GraphPad Prism8 (GraphPad software, La Jolla, CA, USA) was used for statistical testing using two-tailed Student’s *t*-tests and analysis of variance (ANOVA) to determine differences in cell numbers, luciferase activity or immunoprecipitated DNA (* *p* ≤ 0.05; ** *p* ≤  0.01, *** *p*  ≤  0.001). Variance between statistically compared groups was similar.

## 3. Results

### 3.1. Identification of SoxB1-Dependent Changes in the Oligodendroglial Expression Profile

**Figure 1:**
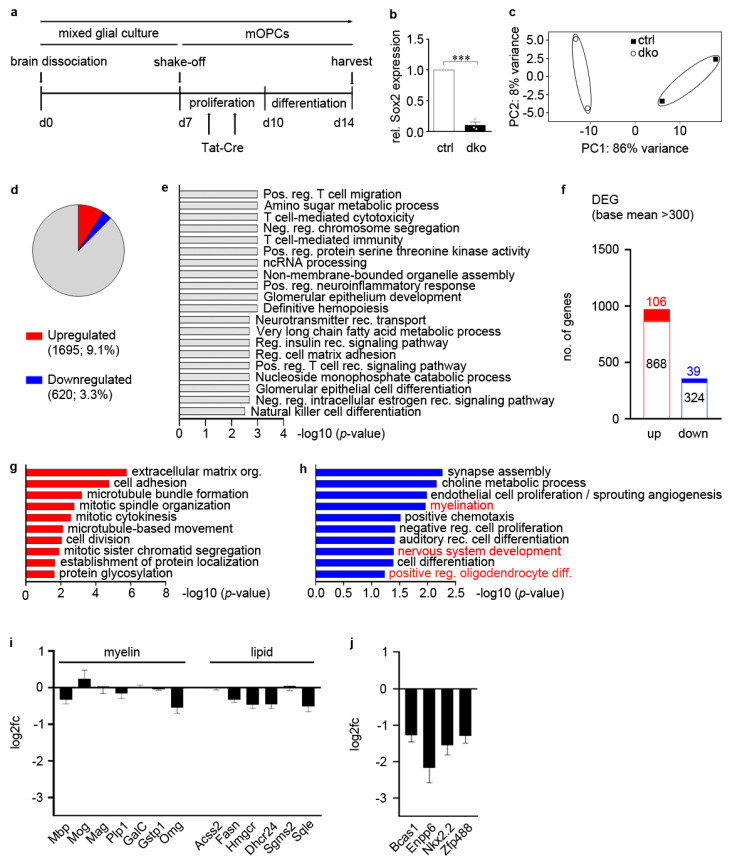
Analysis of SoxB1-dependent expression changes in oligodendroglial cultures after SoxB1 deletion. (**a**) Experimental procedure for obtaining RNA-seq samples. (**b**) Rate of *Sox2* deletion in oligodendroglial cultures from *Sox2^fl/fl^ Sox3^fl/fl^* mice following TAT-Cre application (***, *p* ≤ 0.001). (**c**) PCA plot showing differential clustering of samples from TAT-Cre-treated (dko) and mock-treated (ctrl) oligodendroglial cultures (*n* = 2). (**d**) Pie chart depicting the number of genes with differential expression (DEGs) in TAT-Cre treated oligodendroglial cultures from *Sox2^fl/fl^ Sox3^fl/fl^* mice. The share of upregulated genes is marked in red, the share of downreguated ones in blue as indicated below the pie chart. (**e**) Gene set enrichment analysis (GSEA) of RNA-seq. (**f**) Identification of genes among the DEGs that are expressed (white portion of bars) or even preferentially expressed (filled portion of bars) in oligodendroglial cells. (**g**,**h**) GO term analysis for oligodendroglial-enriched genes upregulated (**g**) or downregulated (**h**) in TAT-Cre-treated oligodendroglial cultures. Terms are sorted by *p*-value. GO terms for downregulated genes discussed in the results are highlighted in red. (**i**,**j**) Expression of myelin genes (**i**), genes associated with lipid metabolism (**i**) and genes associated with the premyelinating state (**j**) in TAT-Cre-treated oligodendroglial cultures from *Sox2^fl/fl^ Sox3^fl/fl^* mice relative to mock-treated controls shown as log2-fold change.

To obtain the first indication of the role of Sox2 and Sox3 in oligodendroglial cells on a mechanistic level, we generated mixed glial cultures from Sox2^fl/fl^ Sox3^fl/fl^ mouse pups and isolated oligodendroglial progenitor cells (mOPCs) by shake-off (Figure 1a). The resulting mOPC cultures were kept for 3 days under proliferating conditions and treated twice during this period with TAT-Cre protein. Subsequently, cells were switched to differentiating culture conditions and kept there for 4 days before harvesting. Mock-treated cultures served as control. This approach was chosen because one of our earlier studies [11] failed to detect dramatic changes in OPC properties or numbers in mice with combined oligodendroglial deletion of Sox2 and Sox3 and found defects restricted to the early stages of oligodendrocyte differentiation. By this protocol, deletion rates of 90% were achieved (Figure 1b). After 4 days in differentiating conditions, cells were harvested and two samples for each treatment underwent RNA-seq for expression profiling. According to the PCA plot, similarly treated probes clustered separately (Figure 1c). Bioinformatic analysis revealed that 1695 genes were upregulated and 620 genes were downregulated (log2fold change ≥ ±0.7) (Figure 1d). According to GSEA, many changes in gene expression were associated with immune-related terms in these cultures such as positive regulation of T cell migration, T cell-mediated immunity or neuroinflammatory response (Figure 1e).

Oligodendroglial cultures prepared by shake-off from mixed glial cultures contain contaminating microglia and astrocytes, especially when prepared from mice. Therefore, the immune-related changes in the RNA-seq studies likely resulted from deletion of Sox2 and Sox3 in microglia and/or astrocytes, or represent secondary inflammatory responses of these cells to changes induced by loss of Sox2 and Sox3 in oligodendroglial cells. To account for this complication, we filtered the list of upregulated and downregulated genes by removing all genes with predominant expression in microglia and immune cells and little expression in oligodendroglial cells, according to Zhang et al., 2014 [25], and set the base mean count to ≥300. This reduced the number of upregulated genes from 1695 to 974 and the number of downregulated genes from 620 to 363 (Figure 1f). Further filtering of these DEG lists revealed that 106 upregulated and 39 downregulated genes were exclusively expressed in oligodendroglia according to Zhang et al., 2014 [25]. Our DEGs exhibited only a minimal overlap with the genes previously identified as being regulated by Sox2 in a mouse oligodendroglioma model [26]. We also did not detect substantial deregulation of *Rgcc* and *PKCθ*, two previously described Sox2 targets in OPCs [12].

Gene ontology studies on the 106 upregulated oligodendroglial genes revealed an enrichment of genes related to terms of cell adhesion and cell division (Figure 1g). The GO analysis of the 39 downregulated oligodendroglial genes pointed to their involvement in myelination, nervous system development and the positive regulation of oligodendrocyte differentiation (Figure 1h, highlighted in red). These results are in agreement with the previously described roles of Sox2 and Sox3 in OPCs and differentiating oligodendrocytes [11,13,14]. Our RNA-seq studies also showed that the deletion of Sox2 and Sox3 had only mild consequences on the expression levels of genes coding for components of the myelin sheaths and lipid metabolism under our experimental conditions (Figure 1i). Instead, closer inspection of the down-regulated genes drew our attention to *Bcas1*, *Enpp6*, *Nkx2.2* and *Zfp488* (Figure 1j) as four genes prominently associated with the premyelinating oligodendrocyte stage and the onset of the differentiation process [27,28,29,30]. This argues that the previously observed effect of Sox2 and Sox3 on oligodendrocyte differentiation [11] may take place in premyelinating oligodendrocytes and may at least in part be mediated by these factors.

### 3.2. Bcas1 as a Direct Target Gene of SoxB1 Proteins

**Figure 2:**
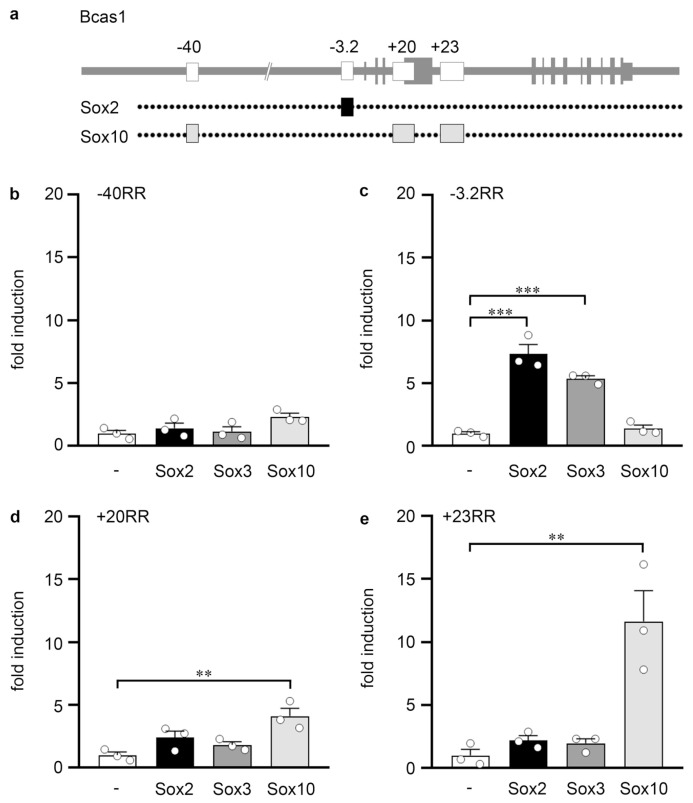
Analysis of Sox-responsive regions in the *Bcas1* locus. (**a**) Scheme of the *Bcas1* genomic locus. Shown at the top is the exon–intron structure of the *Bcas1* gene (vertical lines and boxes in grey correspond to exons) and the localization of the RR (white boxes). Dotted lines below indicate which RR contain Sox2 or Sox10 binding sites according to published ChIP-seq studies for Sox2 in neural precursor cells (GSE35496) and for Sox10 in oligodendroglial cells (GSE64703). (**b**–**e**) Luciferase assays in Neuro2a cells transiently transfected with reporter plasmids carrying the −40RR (**b**), −3.2RR (**c**), +20RR (**d**) and +23RR (**e**) of the *Bcas1* gene in the absence (−) or presence of Sox2, Sox3 or Sox10, as indicated below the bars. Effector-dependent activation rates are presented as fold inductions ± SEM with transfections in the absence of any Sox protein set to 1 (*n* = 3, shown as bullets). Differences were statistically significant as determined by one way ANOVA with Bonferroni correction (**, *p* ≤ 0.01; ***, *p* ≤ 0.001).

To investigate whether Bcas1 is a direct transcriptional target of Sox2 and Sox3, we searched previously published ChIP-seq data for Sox2 in neural precursor cells (GEO accession number GSE35496) and for Sox10 and Olig2 in oligodendroglial cells (GEO accession numbers GSE64703 and GSE42447) to identify regulatory regions in or near the *Bcas1* gene and test them for their responsiveness towards Sox2 and Sox3. We identified four such regions with at least one ChIP-seq peak at locations −40 kb (*Bcas1* −40RR), −3.2 kb (*Bcas1* −3.2RR), +20 kb (*Bcas1* +20RR) and +23 kb (*Bcas1* +23RR) relative to the *Bcas1* transcriptional start site (Figure 2a). After insertion of these regions into luciferase reporter plasmids, we analyzed the activity of each region in the absence or presence of SoxB1 proteins following transfection into mouse Neuro2a neuroblastoma cells (Figure 2b–e). Additionally, we included Sox10 in this analysis to compare the activity of Sox2 and Sox3 with a distantly related Sox protein of high relevance in oligodendrocyte differentiation [6]. Among the four analyzed regions, −40RR did not react with increased activity to the presence of any of the tested Sox proteins (Figure 2b). However, −3.2RR responded to Sox2 and Sox3 with increased activity but remained refractory to the presence of Sox10 (Figure 2c). In contrast, Sox10 slightly activated the +20RR and more strongly the +23RR (Figure 2d,e). These two regulatory regions were unresponsive to either Sox2 or Sox3. We conclude from these results, that the *Bcas1* gene has separate regulatory regions, of which one mediates SoxB1-dependent transcriptional activation and two others Sox10-dependent transcriptional activation in vitro.

### 3.3. Enpp6 as a Direct Target Gene of SoxB1 Proteins

**Figure 3:**
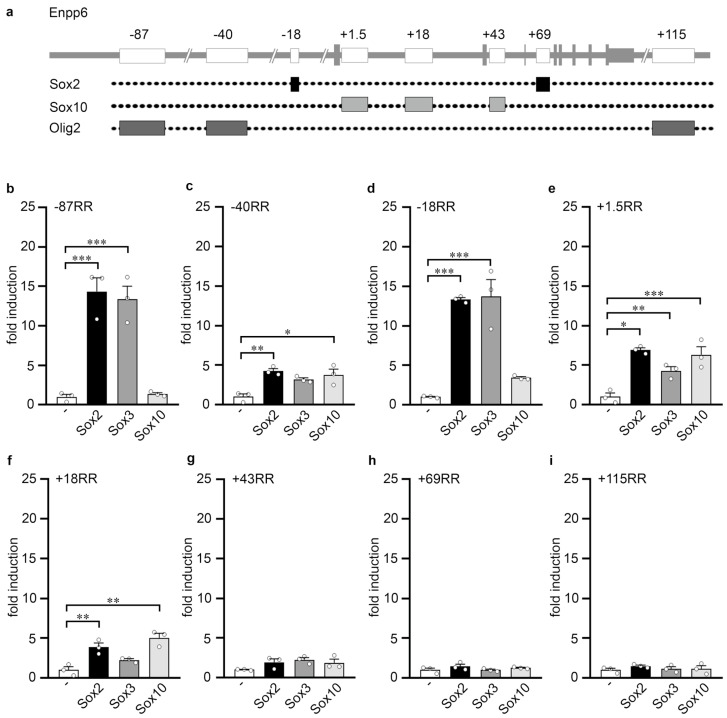
Analysis of Sox-responsive regions in the *Enpp6* locus. (**a**) Scheme of the *Enpp6* genomic locus. Shown at the top is the exon–intron structure of the *Enpp6* gene (vertical lines and boxes in grey correspond to exons) and the localization of the RR (white boxes). Dotted lines below indicate which RR contain Sox2, Sox10 or Olig2 binding sites according to published ChIP-seq studies for Sox2 in neural precursor cells (GSE35496) and for Sox10 and Olig2 in oligodendroglial cells (GSE64703 and GSE42447). (**b**–**i**) Luciferase assays in Neuro2a cells transiently transfected with reporter plasmids carrying the −87RR (**b**), −40RR (**c**), −18RR (**d**), +1.5RR (**e**), +18RR (**f**), +43RR (**g**), +69RR (**h**) and +151RR (**i**) of the *Enpp6* gene in the absence (-) or presence of Sox2, Sox3 or Sox10, as indicated below the bars. Effector-dependent activation rates are presented as fold inductions ± SEM with transfections in the absence of any Sox protein set to 1 (*n* = 3, shown as bullets). Differences were statistically significant as determined by one way ANOVA with Bonferroni correction (*, *p* ≤ 0.05; **, *p* ≤ 0.01; ***, *p* ≤ 0.001).

Analysis of the *Enpp6* locus identified three regulatory regions in front of the transcriptional start site (*Enpp6* −87RR, *Enpp6* −40RR, *Enpp6* −18RR), four intronic regions (*Enpp6* +1.5RR, *Enpp6* +18RR, *Enpp6* +43RR, *Enpp6* +69RR) and one behind the gene (*Enpp6* +151RR) (Figure 3a). Except for the three most distal downstream regulatory regions, all were responsive towards Sox2, and some of them also towards Sox3 (Figure 3b–f). Three of the regulatory regions (i.e., −40RR, +1.5RR, +18RR) exhibited only mild activation rates that were furthermore comparable between the two SoxB1 proteins and Sox10 (Figure 3c,e,f). In contrast, *Enpp6* −87RR and −18RR were more robustly activated by Sox2 and Sox3 and also exhibited higher SoxB1-dependent than Sox10-dependent activation rates (Figure 3b,d). We conclude from these in vitro experiments that Sox2 and Sox3 are likely to exert their effects on *Enpp6* expression predominantly via the −87RR and −18RR regulatory region.

### 3.4. Zfp488 and Nkx2.2 as Direct Target Genes of SoxB1 Proteins

**Figure 4:**
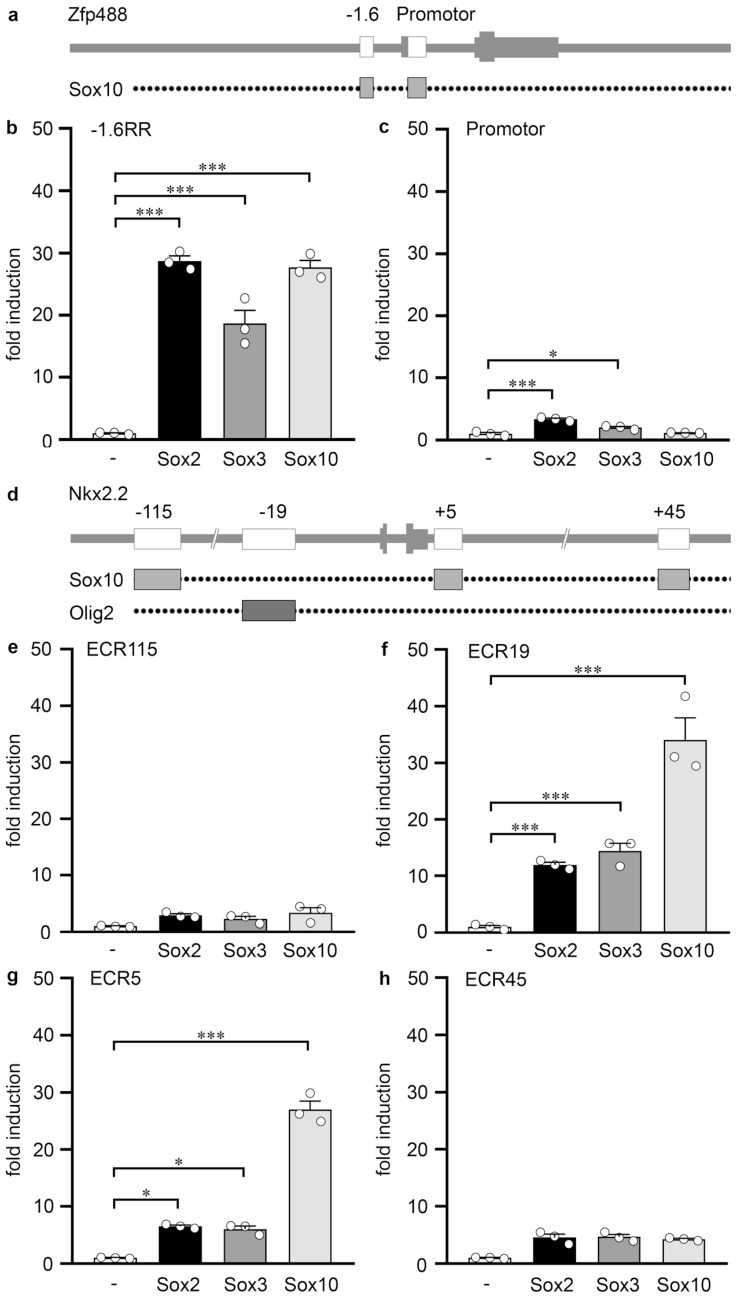
Analysis of Sox-responsive regions in the *Zfp488* and *Nkx2.2* loci. (**a**,**d**) Scheme of the *Zfp488* (**a**) and the *Nkx2.2* (**d**) genomic loci. Shown are introns and exons of the genes (vertical lines and boxes in grey correspond to exons), and the regulatory regions. Dotted lines below the genomic representation indicate which RR contain Sox10 or Olig2 binding sites according to published ChIP-seq studies. (**b**,**c**,**e**–**h**) Luciferase assays in Neuro2a cells transiently transfected with reporter plasmids carrying the −1.6RR (**b**) and the promoter (**c**) of the *Zfp488* gene, as well as ECR115 (**e**), ECR19 (**f**), ECR5 (**g**) and ECR45 (**h**) of the *Nkx2.2* gene in the absence (-) or presence of Sox2, Sox3 or Sox10, as indicated below the bars. Effector-dependent activation rates are presented as fold inductions ± SEM with transfections in the absence of any Sox protein set to 1 (*n* = 3, shown as bullets). Differences were statistically significant, as determined by one way ANOVA with Bonferroni correction (*, *p* ≤ 0.05; ***, *p* ≤ 0.001).

For the *Zfp488* gene, only the promoter and a region −1.6 kb upstream of the transcriptional start site (*Zfp488* −1.6RR) were identified as potential regulatory regions (Figure 4a). Transfection results in Neuro2a cells identified the −1.6RR region as primarily responsive to Sox2 and Sox3 (Figure 4b), whereas the gene promoter did not change its activity drastically in the presence of either protein (Figure 4c). In the case of the −1.6RR, Sox10 was equally active as Sox2 and Sox3, arguing that this particular regulatory region is strongly responsive to both groups of Sox proteins.

In the *Nkx2.2* locus, four potential regulatory regions had been previously localized and tested for their Sox10-responsiveness [17]. They were named ECR115, ECR19, ECR5 and ECR45 (Figure 4d). Among them, only ECR19 and ECR5 mediated a strong reporter gene activation in the presence of Sox10. When tested for their response towards Sox2 and Sox3, the two regulatory regions showed a weaker reporter gene activation (Figure 4f,g), while ECR115 and ECR45 did not react to any of the Sox proteins (Figure 4e,h).

### 3.5. Sox2 Binding to Responsive Regulatory Regions and Interaction with Transcription Factors Relevant for Premyelinating Oligodendrocytes

**Figure 5:**
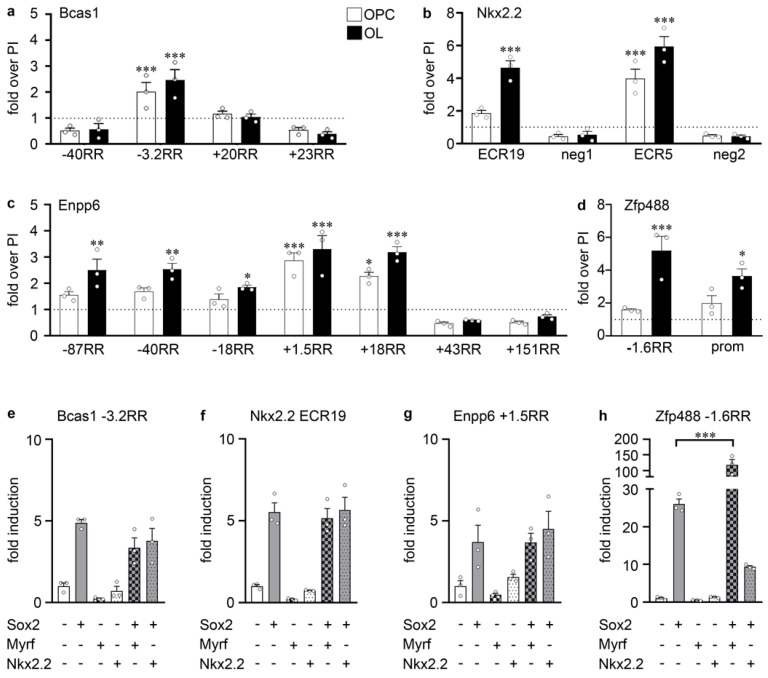
Molecular action of Sox2 on its responsive regions. (**a**–**d**) ChIP experiments on primary rat oligodendroglial cells. Chromatin from OPCs (white bars) or OLs (black bars) was precipitated with an anti-Sox2 antiserum and the corresponding pre-immune serum to detect Sox2 occupancy on Sox2-responsive regions from the *Bcas1* (**a**), *Nkx2.2* (**b**), *Enpp6* (**c**) and *Zfp488* (**d**) genes as compared to negative control regions. Amounts of precipitated chromatin were determined as fold over enrichment obtained by pre-immune serum (set to 1, see dotted line). (**e**–**h**) Luciferase assays in Neuro2a cells transiently transfected with reporter plasmids carrying the *Bcas1* −3.2RR (**e**), the *Nkx2.2* ECR19 (**f**), the *Enpp6* +1.5RR (**g**) or the *Zfp488* −1.6RR (**h**) in the absence (-) or presence (+) of Sox2, Nkx2.2, Myrf or combinations thereof, as indicated below the bars. Effector-dependent activation rates are presented as fold inductions ± SEM with transfections in the absence of any Sox protein set to 1 (*n* = 3, shown as bullets). Differences were statistically significant as determined by one way ANOVA with Bonferroni correction (*, *p* ≤ 0.05; **, *p* ≤ 0.01; ***, *p* ≤ 0.001).

Considering that some of the regulatory regions that mediate a Sox2 and a Sox3 response were originally identified as binding regions for other transcription factors, such as Olig2 and Sox10, or have been described as Sox2 binding in neural precursor cells but not oligodendroglial cells, we next performed chromatin immunoprecipitation experiments with anti-Sox2 antibodies. We investigated whether the Sox2-responsive regions of *Bcas1* (−3.2RR), *Enpp6* (−87RR, −40RR, −18RR, +1.5RR, +18RR) and *Zfp488* (−1.6RR, promoter) as well as the evolutionary conserved regions of the *Nkx2.2* gene (ECR19, ECR5) were preferentially precipitated from oligodendroglial chromatin with antiserum directed against Sox2 as compared to the pre-immune control. Despite variation in the exact level, all Sox2-responsive regions were significantly enriched in the precipitate (Figure 5a–d). In contrast, other regions from the *Bcas1* (−40RR, +20RR, +23RR) and the *Enpp6* (+43RR, +151RR) genes that did not respond to Sox2 in our transfection analyses also failed to become enriched in the Sox2-precipitated oligodendroglial chromatin. In most cases, enrichment after Sox2-mediated precipitation was at least slightly stronger with chromatin from differentiated oligodendrocytes than with OPC chromatin. These findings argue that the identified Sox2-responsive regulatory regions of the *Bcas1*, *Enpp6*, *Zfp488* and *Nkx2.2* genes are likely also bound by Sox2 in vivo (Figure 5a–d).

The onset of *Bcas1*, *Enpp6*, *Zfp488* and *Nkx2.2* expression in premyelinating oligodendrocytes furthermore correlates with the appearance of Myrf and Nkx2.2 as two transcription factors responsible for oligodendroglial development in this particular stage [29,31]. Therefore, we analyzed whether we could obtain any evidence for the cooperation of Sox2 with either Myrf or Nkx2.2 in the regulation of *Bcas1*, *Enpp6*, *Zfp488* and *Nkx2.2* expression in reporter gene assays (Figure 5e–h). However, co-transfection of Sox2 with either Myrf or Nkx2.2 did not lead to an increased transcriptional activation of the Sox2-responsive *Bcas1*, *Nkx2.2* or *Enpp6* regulatory regions that was substantially higher than the activation obtained with Sox2 alone. Figure 5e–g shows the results for *Bcas1* −3.2RR, *Nkx2.2* ECR19 and *Enpp6* +1.5RR as examples. Only the *Zfp488* −1.6RR responded in a marked way towards the joint presence of Sox2 and Myrf and exhibited a robust 118-fold activation as compared to a 28-fold activation observed for Sox2 and absent activation in the presence of Myrf alone (Figure 5h). *Zfp488* −1.6RR is thus synergistically activated by Sox2 and Myrf.

### 3.6. Expression of Bcas1, Enpp6, Zfp488 and Nkx2.2 in SoxB1-Deficient Oligodendroglial Cells In Vivo

The data presented so far support the notion that several genes closely associated with the premyelinating stage in oligodendrocytes are under direct transcriptional control of Sox2 and Sox3. To further validate and verify this conclusion in vivo, we used mice that, within their CNS, specifically lack Sox2 and Sox3 in oligodendroglial cells by combining *Sox2^fl^* and *Sox3^fl^* alleles with a Sox10-Cre BAC transgene [9]. It has been shown that in these double conditional knockout (dko) mice, deletion of both genes in oligodendroglial cells is in the range of 94–97% at E15.5, and that these mice do not survive beyond birth.

We prepared transverse spinal cord sections from dko mice at birth and analyzed them in comparison to age-matched control sections for expression of Bcas1, Enpp6, Zfp488 and Nkx2.2 by immunohistochemistry and in situ hybridization. It has previously been shown that the spinal cord expression of all four genes is restricted to oligodendroglial cells at this age [Fard, 2017 #2739; Xiao, 2016 #2800; Wang, 2006 #1425; Ref. [26].

**Figure 6:**
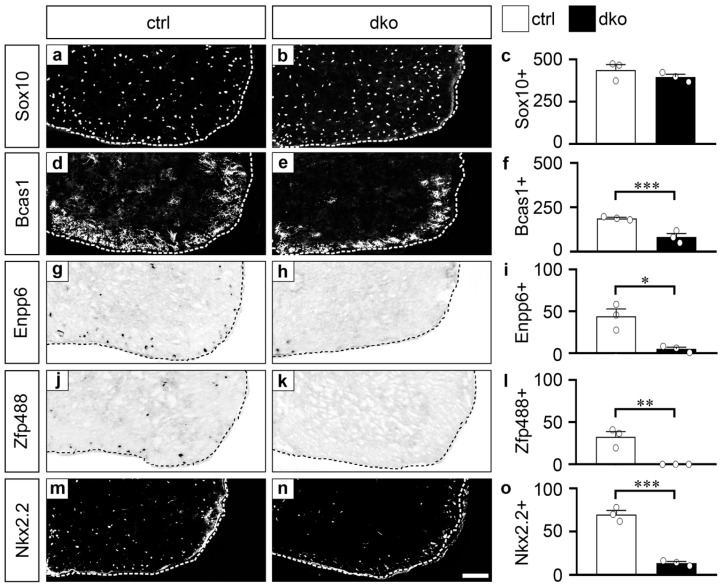
Oligodendroglial expression of Bcas1, Enpp6, Zfp488 and Nkx2.2 in SoxB1 mouse mutants. (**a**–**f**,**m**–**o**) Immunohistochemical staining of transverse thoracic spinal cord sections of newborn wildtype (ctrl; (**a**,**d**,**m**)) and Sox2^fl/fl^ Sox3^fl/fl^ Sox10-Cre (dko) mice (**b**,**e**,**n**) using antibodies directed against Sox10 (**a**,**b**), Bcas1 (**d**,**e**) and Nkx2.2 (**m**,**n**) as well as quantifications of marker-positive cell numbers per section (**c**,**f**,**o**) as mean values ± SEM (*n* = 3 spinal cord specimen, three sections per spinal cord counted in full) from these stainings. (**g**–**l**) In situ hybridization of spinal cord tissue from newborn wildtype (**g**,**j**) and dko (**h**,**k**) mice using antisense riboprobes specific for Enpp6 (**g**,**h**) and Zfp488 (**j**,**k**) and resulting quantifications (**i**,**l**) of transcript-positive cells as mean values ± SEM (*n* = 3 spinal cord specimen, marked by bullets; three sections per spinal cord counted in full). Scale bar: 100 µm (**n**). Statistical analysis was performed with Student’s two-tailed *t*-test (* *p* ≤ 0.05, ** *p* ≤ 0.01, *** *p* ≤ 0.001).

Immunohistochemical staining with antibodies directed against Sox10 confirmed that the overall oligodendroglial numbers were not significantly affected by the loss of Sox2 and Sox3 (Figure 6a–c). However, the number of Bcas1-positive premyelinating oligodendrocytes was more than halved (82.0 ± 19 as compared to 188 ± 5 in controls; Figure 6d–f). Similarly, there was a dramatic reduction of the *Enpp6*-positive and the *Zfp488*-positive cells in the spinal cord of dko mice, as evidenced by in situ hybridization (Figure 6g–l). The Nkx2.2-expressing oligodendroglial cells were also reduced to 19% in dko mice according to immunohistochemical staining for Nkx2.2 (Figure 6m–o). Through the use of antibodies for Bcas1 and Nkx2.2, we confirmed that the expression in dko mice was not only reduced on the transcript but also on the protein level. For Enpp6 and Zfp488, only transcript levels could be studied because none of the commercially available antibodies that we tested worked reliably in our hands. Despite this, the reduction in the number of positive cells for all four markers strongly supports the notion that the oligodendroglial expression of all four genes is under the control of Sox2 and Sox3 in vivo.

## 4. Discussion

In this study, we analyzed the consequences of a combined loss of Sox2 and Sox3 on the oligodendroglial expression profile. Interestingly, we found evidence for downregulation of factors that are relevant for establishing the premyelinating stage. We focused our analysis on *Bcas1*, *Enpp6*, *Zfp488* and *Nkx2.2*. These genes are downregulated in Sox2/Sox3-deficient oligodendroglial cells and are also strongly associated with the premyelinating stage during developmental myelination as well as remyelination [27,28,29,30].

To investigate whether *Bcas1*, *Enpp6*, *Zfp488* and *Nkx2.2* constitute direct target genes of Sox2 and Sox3, we used previously published ChIP-seq data to select potential SoxB1-responsive regulatory regions. We were able to identify at least one region for each of the four genes that responded in transient transfection assays to Sox2 and Sox3. Further validation by ChIP-PCR confirmed that each of the Sox2/Sox3-responsive regions was indeed bound by Sox2 in oligodendroglial cells. This strengthens the assumption that the identified regions serve as bona fide Sox2-responsive elements in vivo. Further in vivo support for a direct effector–target gene relationship between Sox2 and Sox3 on the one and the *Bcas1*, *Enpp6*, *Zfp488* and *Nkx2.2* genes on the other side comes from the observation that all four factors are downregulated in oligodendroglial cells of newborn mice that lack Sox2 and Sox3. Considering the role of Bcas1, Enpp6, Zfp488 and Nkx2.2 in premyelinating oligodendrocytes, it appears reasonable to assume that Sox2 exerts its effects on terminal oligodendrocyte differentiation at least in part through these factors during the premyelinating stage.

When analyzing the potential regulatory regions for their responsiveness towards Sox2 and Sox3, we also tested their ability to react to the presence of Sox10. In the case of the *Bcas1* gene, Sox2 and Sox3 appear to act through a completely different regulatory region than Sox10. For *Enpp6*, the two most responsive regulatory regions have a clear preference for Sox2 and Sox3 as compared to Sox10, while the single regulatory region from the *Zfp488* gene exhibits comparable responsiveness to Sox2, Sox3 and Sox10. In the case of *Nkx2.2*, Sox2 and Sox3 use the same regulatory regions as Sox10 but appear to be less effective. We interpret these data in such a way that *Bcas1*, *Enpp6*, *Zfp488* and *Nkx2.2* are not only target genes of Sox2 and Sox3 but also of Sox10. Whether regulatory regions are shared between Sox2 and Sox3 on the one hand and Sox10 on the other hand, appears to vary from gene to gene. Reasons for regulation by SoxB1 proteins as well as Sox10 are manifold. SoxB1 proteins and Sox10 may each contribute to the overall activation rate in premyelinating oligodendrocytes. Alternatively, Sox2 and Sox3 could be primarily responsible for activation in premyelinating oligodendrocytes, and Sox10 may take over at later stages once the expression of Sox2 and Sox3 has subsided.

It is intriguing that Sox2 and Sox3 appear to exert their effect on the expression of several genes through different regulatory regions than Sox10. This is in agreement with findings that distantly related Sox proteins (i.e., members of different subgroups of the Sox family) usually use distinct cis-acting regulatory elements and selective interactions with transcriptional partner proteins to exert their effects on target gene expression (for review, see Refs. [15,32,33]). Even in cases where the same regulatory region mediates the effects of SoxB1 proteins and Sox10, different cis-acting elements may be involved.

In this respect, it will be interesting to determine the proteins that Sox2 and Sox3 interact with during the activation of the *Bcas1*, *Enpp6*, *Zfp488* and *Nkx2.2* genes in premyelinating oligodendrocytes. One potential candidate for such a partner is Myrf, as its expression starts in premyelinating oligodendrocytes under the control of Sox10 [16,31]. Another one is Nkx2.2 [29]. Interestingly, we were able to identify Myrf as a functional interaction partner for Sox2 on the *Zfp488* −1.6RR. However, none of the other major Sox2/Sox3-responsive regions showed any signs of cooperative activation when Myrf or Nkx2.2 were present in addition to Sox2 or Sox3. While we cannot exclude that a common interaction partner for Sox2 and Sox3 exists for activation of *Bcas1*, *Enpp6*, *Zfp488* and *Nkx2.2*, it may well be that Sox2 and Sox3 rely on different interaction partners for each gene. In this context, it deserves to be mentioned that Olig1 has been described as an activator of *Zfp488* expression in oligodendroglial cells [30]. In a similar vein, Olig2, Sox10 and Nfat proteins have been identified as activators of *Nkx2.2* expression [17,29,34,35]. It will therefore be interesting to test in future experiments whether Olig or Nfat proteins cooperate with Sox2 and Sox3 in premyelinating oligodendrocytes during the activation of select genes. Combinations with transcription factors that only become active or start to be present in premyelinating oligodendrocytes could also explain how Sox2 and Sox3 are able to activate specific genes at this developmental stage in addition to the genes they activate earlier on in OPCs.

Despite the remaining open questions, our study helps to explain the role of Sox2 and Sox3 during oligodendrocyte differentiation and unravels at least part of its mode of action by identifying relevant downstream effectors.

## Data Availability

All data generated or analyzed during this study are included in this published article or were deposited in GEO under accession number GSE263925 [https://www.ncbi.nlm.nih.gov/geo/query/acc.cgi?acc=GSE263925].
